# Sexual violence against women in the Western Amazon

**DOI:** 10.11606/s1518-8787.2021055003069

**Published:** 2021-11-16

**Authors:** Júlia Souza Santos Cargnin, Juliana Scholtão Luna, Débora Melo de Aguiar, Bárbara Teles Cameli Rodrigues, Aldir Alves de Azevedo, Rodrigo Pinheiro Silveira

**Affiliations:** I Universidade Federal do Acre Programa de Pós-Graduação em Saúde Coletiva Rio Branco AC Brasil Universidade Federal do Acre. Programa de Pós-Graduação em Saúde Coletiva. Rio Branco, AC, Brasil; II Universidade Federal do Acre Centro de Ciências da Saúde e do Desporto Rio Branco AC Brasil Universidade Federal do Acre. Centro de Ciências da Saúde e do Desporto. Rio Branco, AC, Brasil; III Universidade Federal do Acre Programa de Pós-Graduação em Saúde Coletiva Rio Branco AC Brasil Universidade Federal do Acre. Programa de Pós-Graduação em Saúde Coletiva. Rio Branco, AC, Brasil

**Keywords:** Child Abuse, Rape, Pregnancy in Adolescence, Violence Against Women

## Abstract

**OBJECTIVE:**

To characterize sexual violence cases suffered by women notified by the Sistema de Informação de Agravos de Notificação (Information System for Notifiable Diseases) in the city of Rio Branco (AC - Brazil) from 2011 to 2016.

**METHODS:**

Descriptive study based on information from the Notifiable Diseases Information System. The study population consisted on women victims of sexual violence reported in the city of Rio Branco (AC), from 2011 to 2016.

**RESULTS:**

The results show a higher number of notifications during 2012, especially among single, brown, and aged between 10 and 14 years victims; usually they have 5 to 8 years of schooling. Normally violence occurred in residences and by a single aggressor, male and non-alcoholic.

**DISCUSSION:**

The large number of notifications of pregnant women aged 10 to 14 years corresponds to the compulsory notification of rape of a vulnerable person, identified at the time of prenatal care or childbirth.

**CONCLUSION:**

We confirm the susceptibility to sexual violence of young women in Rio Branco, raising the issue of child marriage and teenage pregnancy.

## INTRODUCTION

Sexual violence (SV) is one kind of violence practiced against women and is considered one of the cruelest and persistent throughout history. Defined as any violent act or attempt to obtain sex, or against the person’s will, regardless of the type of interpersonal relationship that exists. Sexual violence includes verbal aggression, economic advantage, forced marriage, sexual harassment, and rape^1^.

Some authors claim that factors such as age, education, income, alcohol and/or drug use, marital status, and sexual orientation predispose the victim to sexual violence. However, the real prevalence is difficult given underreporting, caused by embarrassment or fear of the victim or given the variability between studies in relation to populations, instruments, privacy conditions, training of interviewers, techniques for collecting information, and the definitions of violence adopted in the methodology^2^.

However, reliable indicators have shown that SV is among the main causes of the reduction in years of healthy life due to disabilities^3^. According to Schraiber^4^, SV follows a common pattern, occurring mainly in private environments, perpetrated by intimates and cyclically repeated.

In the last decades, Brazil improved some strategies to prevent SV against women, implementing the *Delegacias Especializadas de Atendimento às Mulheres* (Specialized Police Stations for Assistance to Women), *Coordenadorias das Mulheres* (Women’s Coordination), *Políticas de Enfrentamento à Violência* (Policies to Combat Violence) and the *Lei Maria da Penha* (Maria da Penha Law).

Since 2004, health services are responsible for the SV and domestic compulsory notification. However, just five years later, with the *Sistema de Vigilância de Violências e Acidentes* (VIVA - Violence and Accident Surveillance System), connected with the *Sistema Informações de Agravo de Notificações* (SINAN - Notifications System Information), the notification of interpersonal and self-inflicted violence is carried out in an incipient way, in sentinel units of the municipalities^5^. Since 2014, both SV and domestic violence are immediately notified. Therefore, the SINAN started to be used as a standardized instrument for data collection and registration, making it an important database system for epidemiological studies and planning of health actions^6^. However, women should decide on the registration of the police report and the performance of expert examinations.

Even taking into account underreporting, official data from the Ministry of Health (MH) for 2011 indicated 15.9 cases of SV per 100,000 women, with variations according to the Brazilian regions examined and socioeconomic and educational levels of the population^7^.

The scarcity of epidemiological studies that address the issue of SV against women in the North of the country is possibly because of the vast territorial extension and the existence of locations of difficult access, some lacking essential public services, added to the precarious socioeconomic conditions of some population – moreover, the development of prevalence studies is laborious and the underreporting of SV cases is a serious reality^8^.

This situation is similar in the municipality of Rio Branco, capital of the state of Acre. Therefore, this article seeks to characterize the cases of SV against women reported in the SINAN in the municipality, considering the period from 2011 to 2016.

## METHODS

This is an observational and descriptive study, based on a retrospective and quantitative approach, which analyzes the profile of SV against women notified in health services from 2011 to 2016 in the city of Rio Branco (AC - Brazil).

The study used data from Sistema de Informação de Agravos de Notificação (Information System for Notifiable Diseases) regarding notifications in the municipality of Rio Branco, based on Ficha de Notificação (FIN - Notification Form) of domestic, sexual and/or other violence, provided by the Secretaria de Saúde Municipal (Municipal Health Department).

Almost all notifications, both in the city and in the state, are carried out at the Maternity Bárbara Heliodora (MBH), a public health unit located in the capital and a reference in the care of victims of SV and the only registered for the procedure of legal termination of pregnancy. The MBH provides multidisciplinary assistance to women victims of SV. The maternity has a trained team to carry out medical and multidisciplinary procedures, also dealing with the necessary referrals in the support network.

In the period from 2011 to 2016, 3,358 FIN for domestic, sexual and/or other violence were filled. Of these, 1,648 cases corresponded to SV against women aged 10 years or over. The MBH was responsible for the notification of 1,529 cases; therefore, 92.8% of the registered notifications.

For the present study, all cases with a record of notification of interpersonal and self-inflicted violence with the generic code Y09 were selected from the SINAN database, which corresponds to aggressions by unspecified means according to the International Statistical Classification of Diseases and Related Health Problems (CID) – 10. Besides, they were all women. As inclusion criteria for cases, the confirmation of the completion of the “sexual violence” field and the victim’s age equal to or greater than 10 years were used.

Therefore, the study population for epidemiological characterization consisted of suspected or confirmed cases of SV, female and aged 10 years or over, reported in the municipality of Rio Branco, from January 1, 2011 to December 31 2016. We analyzed the 1,648 cases that met the criteria.

Sociodemographic information was also collected, such as age group, marital status, race, and education. They are all related to the woman’s condition, for example, pregnant and/or with the presence of a disability or disorder; information on violence, such as place of occurrence, time, repetition of cases, associated violence, type of penetration, number of people involved, gender of the likely aggressor, suspected alcohol use, bond with the victim, physical injuries and other consequences; and referrals taken, for example, STD prophylaxis, semen collection, emergency contraception, the procedure for legal abortion, referral to the *Delegacia da Mulher* (Women’s Police Station), referral to the *Conselho Tutelar* (Guardian Council), referral to the *Delegacia de Proteção à Criança* (Child Protection Police) and referral to the health services.

Considering the national legislation on the rape of the vulnerable (< 14 years old), a separate analysis was performed in age groups, being classified as vulnerable victims between 0 and 14 years old; young victims between 15 and 19 years old; and adult victims between 20 or more. Subsequently, it was dichotomized into pregnant women aged 10 to 14 years and pregnant women aged 15 or over, identifying a higher incidence of cases in the latter group.

For categorical variables, absolute (n) and relative (%) frequencies were obtained, and continuous variables were analyzed using measures of central tendency (mean and median) and measures of dispersion (standard deviation). Statistical differences between groups were verified using the chi-square test, considering a significance level of 5%. We used the Statistical Package for Social Science (SPSS) version 20 to proceed data analysis. This study respects the ethical principles of research with human beings, having been approved by the Ethics and Research Committee of the HCA/FUNDHACRE under process n° 2.127.216.

## RESULTS

During the period examined, the distribution of SV notifications against women in the city of Rio Branco shows a higher concentration of cases between the years 2012 (18.7%) and 2013 (19.2%) (data not shown in table).

Table 1 describes the epidemiological profile of women victims of SV between 2011 and 2016, with a mean age of 15.56 years, median of 16 years, minimum age of 10 and maximum of 65. Regarding the frequency distribution, the largest proportion of women was between 10 and 14 years old (70.8%). They were single (68.5%), brown (84.0%), had between 5 and 8 years of formal education (60.3%), were pregnant (51.8%), had no disability or disorder (95.3%), and lived in Rio Branco (73.4%).

**Table 1:** Frequency distribution in the studied variables referring to the victim, the type of aggression and the aggressor in Rio Branco (AC) from 2011 to 2016.

Variable		n	%
**Age group**			
	10 to 14 years	1,166	70.8
	15 to 19 years	268	16.2
	20 years old or more	214	13.0
	Total	1,648	100.0
**Marital Status**			
	Single	1,061	68.5
	Married/common-law marriage	473	30.6
	Separated/Widow	14	0.9
	Total	1,548	100.0
**Ethnicity/Race**			
	White	157	9.6
	Black	76	4.6
	Brown	1,376	84.0
	Yellow/Indigenous	30	1.8
	Total	1,639	100.0
**Formal education**			
	Up to 4 years of study	262	16.6
	5 to 8 years of study	955	60.3
	9 to 11 years of study	321	20.3
	12 years or more of study	45	2.8
	Total	1,583	100
**Pregnant**			
	Yes	771	51.8
	No	717	48.2
	Total	1,488	100
**Presence of disability or disorder**			
	Yes	76	4.7
	No	1,554	95.3
	Total	1,630	100
**City of occurrence**			
	Rio Branco	1,147	73.4
	Others	416	26.6
	Total	1,563	100
**Occurrence location**			
	Residence	1,350	84.5
	Street	101	6.3
	Collective housing	27	1.7
	School	10	0.6
	Trade/Services	8	0.5
	Industries/Construction	7	0.4
	Bar or similar	4	0.3
	Sports practice venue	2	0.1
	Other/Motel	88	5.5
	Total	1,597	100
**Time of occurence**			
	00h01–06h00	141	11.6
	06h01–12h00	189	15.6
	12h01–18h00	239	19.7
	18h01–24h00	645	53.1
	Total	1,214	100
**Happened other times**			
	Yes	1,049	66.0
	No	541	34.0
	Total	1,590	100.0
**Associated violence**			
	No	1,200	73.2
	Threat	26	1.6
	Body strength	63	3.8
	White Arms	14	0.9
	Hanging	4	0.2
	Firearm	112	6.8
	Injury by sharp object	1	0.1
	Burn	1	0.1
	More than one type	204	12.4
	Others	14	0.9
	Total	1,639	100
**Type of penetration**			
	No penetration	31	2.8
	Oral	10	0.9
	Anal	12	1.1
	Vaginal	949	85.7
	More than one type	105	9.5
	Total	1,107	100
**Number of involved**			
	One	1,538	94.0
	Two or more	99	6.0
	Total	1,637	100
**Gender of the likely offender**			
	Male	1,606	97.7
	Female	29	1.8
	Both sexes	9	0.5
	Total	1,644	100.0
**Suspicion of alcohol use**			
	Yes	415	26.3
	No	1,164	73.7
	Total	1,579	100.0
**Bond with the victim**			
	Intimate partner	874	53.7
	Known	315	19.3
	Family	103	6.3
	Unknown	266	16.3
	Others	71	4.4
	Total	1,629	100.0

The violence occurred in residence in 84.5% of cases. The violence occurred mainly at night, between 6:01 pm and midnight, representing 53.1%. 66.0% of the cases were recidivists and 73.2% were not associated with other violence. As for the violent act and the aggressor, most cases were reported vaginal penetration (85.7%), only one aggressor involved (94.0%), male (97.7%), with no suspicion regarding the consumption of alcohol (73.7%). Victims were violented by intimate partners in most cases (53.7%).

Table 2 depicts the profile of SV victims according to sociodemographic characteristics and age. There was a predominance of women aged 10 to 14 years, mixed-race, between 5 and 8 years of formal education, and single, with statistically significant variation between age groups.

**Table 2:** Characteristics of women victims of sexual violence by age group, according to sociodemographic characteristics, reported in Rio Branco (AC) from 2011 to 2016.

Characteristic		Total		10 to 14 years		15 to 19 years		20 or more		p
		n	(%)	n	(%)	n	(%)	n	(%)	
**Skin color**										
	White	157	9.6	85	7.3	36	13.5	36	17.0	0.000^a^
	Black	76	4.6	53	4.6	12	4.5	11	5.2	
	Brown	1,376	83.6	996	85.9	217	81.3	163	76.9	
	Yellow	5	0.3	3	0.3	0	0	2	0.9	
	Indigenous	25	1.5	23	2.0	2	0.7	0	0.0	
**Education**										
	0–4	262	16.6	204	18.2	21	8.1	37	18.1	0.000^a^
	5–8	955	60.3	788	70.4	121	46.7	46	22.5	
	9–11	321	20.3	128	11.4	104	40.2	89	43.6	
	12 or more	45	2.8	0	0.0	13	5.0	32	15.7	
**Occupation**										
	Student	665	66.0	495	71.3	141	74.2	29	23.4	0.000^a^
	Housekeeper	22	2.2	0	0.0	5	2.6	17	13.7	
	Others	321	31.8	199	28.7	44	23.2	78	62.9	
**Marital status**										
	Single	1,061	68.5	727	67.6	206	78.3	128	61.2	0.000^a^
	Married/common-law marriage	473	30.6	346	32.2	57	21.7	70	33.5	
	Divorced	9	0.6	2	0.1	0	0.0	7	3.4	
	Widow	5	0.3	1	0.1	0	0.0	4	1.9	

Ignored or blank values: Race: 0.9%; Education: 1.9%; Occupation: 19.1%; Marital status: 1.1%.

a p-value < 0.05 indicating statistically significant difference by the chi-square test.

Table 3 presents the characteristics of the violent act and the aggressor according to the victim’s age group. Regardless of age, the residence was the most reported place of occurrence. The night shift was mainly reported, except among women aged 20 or over, wherein the highest occurrence was during dawn. Violence was predominantly caused by the intimate partner among the younger women and by strangers among women aged 20 and over. The violent act was committed by only one aggressor, more than once among minors and without an alcoholic history. The main type of penetration was vaginal, with no records of physical injuries. However, the latter case was associated with sexual assault in 18.2% of the records, being more frequent among women aged 20 years or more. Pregnancy was the main consequence among the younger ones, while for the other age groups we did not find recorded consequences.

**Table 3:** Characteristics of sexual violence according to aggression and the aggressor in different age groups of women reported in Rio Branco (AC) from 2011 to 2016.

Characteristic		Total		10 to 14 years		15 to 19 years		20 or more		p
		n	%	n	(%)	n	(%)	n	(%)	
**Location of the occurrence**										
	Residence	1,350	84.5	1,037	91.4	185	72.8	128	61.2	0.000^a^
	Street	101	6.3	29	2.6	28	11.0	44	21.1	
	Other	146	9.1	68	6.0	41	16.1	37	17.7	
**Occurrence shift**										
	Evening	645	53.1	494	60.4	91	45.0	60	30.9	0.000^a^
	Afternoon	239	19.7	161	19.7	48	23.8	30	15.5	
	Morning	189	15.6	125	15.3	28	13.9	36	18.6	
	Dawn	141	11.6	38	4.6	35	17.3	68	35.1	
**Bond with the aggressor**										
	intimate partner	874	53.7	761	66.1	91	34.3	22	10.4	0.000^a^
	Known	315	19.3	198	17.2	61	23.0	56	26.4	
	Family	103	6.3	80	6.9	19	7.2	4	1.9	
	Unknown	266	16.3	60	5.2	82	30.9	124	58.5	
	Others	71	4.4	53	4.6	12	4.5	6	2.8	
**Number of involved**										
	One	1,538	94.0	1,120	96.5	240	90.2	178	84.8	0.000^a^
	Two or more	99	6.0	41	3.5	26	9.8	32	15.2	
**Repeat violence**										
	Yes	1,049	66.0	862	76.8	128	49.2	59	28.5	0.000^a^
	No	541	34.0	261	23.2	132	50.8	148	71.5	
**Use of alcohol**										
	Yes	415	26.3	209	18.5	80	31.7	126	63.0	0.000^a^
	No	1,164	73.7	918	81.5	172	68.3	74	37.0	
**Type of penetration**										
	No penetration	31	2.8	25	3.3	2	1.0	4	2.7	0.000^a^
	Vaginal	949	85.7	675	89.5	177	86.8	97	65.1	
	Anal	12	1.1	5	0.7	3	1.5	4	2.7	
	Oral	10	0.9	3	0.4	3	1.5	4	2.7	
	More than one	105	9.5	46	6.1	19	9.2	40	26.8	
**Physical injury**										
	Yes	200	18.2	107	14.2	39	18.9	54	38.0	0.000^a^
	No	900	81.8	645	85.8	167	81.1	88	62.0	
**Consequences**										
	None	481	43.1	243	31.9	117	56.8	121	81.2	0.000^a^
	STD	19	1.7	13	1.7	4	1.9	2	1.3	
	Pregnancy	607	54.3	499	65.5	84	40.8	24	16.1	
	Suicide	2	0.2	1	0.1	0	0.0	1	0.7	
	Two or more	8	0.7	6	0.8	1	0.5	1	0.7	

Ignored or blank values: Occurrence location: 1.5%; Occurrence shift: 12.9%; Type of penetration: 16.1%; Repeat violence: 1.7%; Consequence of violence: 15.8%; Associated physical injury: 16.3%; Number of involved: 0.3%; Bond with the aggressor: 0.6%; Alcohol use by the aggressor: 2.1%.

a p-value < 0.05 indicating statistically significant difference by the chi-square test.

Regarding pregnant victims, regardless of age, the largest proportion of cases were single women, residing in the main place of occurrence of violence, predominantly in the night shift, with the main aggressor being the intimate partner. Violence was recorded as being repeated, with a higher occurrence of vaginal penetration, not associated with physical injury and having pregnancy as the main consequence (Table 4).

**Table 4:** Characteristics of pregnant women victims of sexual violence according to the aggression suffered and the aggressor for pregnant women up to 14 years old and other pregnant women notified in Rio Branco (AC) from 2011 to 2016.

Characteristic		Pregnant women between 10 and 14 years old		Pregnant women aged 15 or over		p
		n	(%)	n	(%)	
**Marital status**						
	Single	368	58.5	79	64.2	0.636
	Married/common-law marriage	259	41.2	44	35.8	
	Divorced	1	0.2	0	0.0	
	Widow	1	0.2	0	0.0	
**Location of the occurrence**						
	Residence	596	94.8	102	85.7	0.000^a^
	Street	6	1.0	7	5.9	
	Others	27	4.3	10	8.4	
**Occurrence shift**						
	Evening	302	70.1	44	52.4	0.000^a^
	Afternoon	70	16.2	18	21.4	
	Morning	54	12.5	15	17.9	
	Dawn	5	1.2	7	8.3	
**Bond with aggressor**						
	Intimate partner	556	87.0	75	60.5	0.000^a^
	Known	61	9.5	14	11.3	
	Family	8	1.3	2	1.6	
	Unknown	7	1.1	29	23.4	
	Others	7	1.1	4	3.2	
**Repeat violence**						
	Yes	523	83.4	77	63.6	0.000^a^
	No	104	16.6	44	36.4	
**Type of penetration**						
	No penetration	2	0.5	0	0.0	0.001^a^
	Vaginal	421	98.4	95	92.3	
	Anal	1	0.2	2	1.9	
	More than one type	4	0.9	6	5.8	
**Physical injury**						
	Yes	55	12.9	11	10.6	0.518
	No	371	87.1	93	89.4	
**Consequence**						
	None	23	5.4	12	11.7	0.069
	Pregnancy	402	93.7	89	86.4	
	Suicide	1	0.2	0	0.0	
	Two or more	3	0.7	2	1.9	

Ignored or blank values: Marital status: 1.1%; Place of occurrence: 1.5%; Occurrence shift: 12.9%; Type of penetration: 16.1%; Repeat violence: 1.7%; Consequence of violence: 15.8%; Associated physical injury: 16.3%; Number of involved: 0.3%; Bond with the aggressor: 0.6%; Alcohol use by the aggressor: 2.1%.

a p-value < 0.05 indicating statistically significant difference by the chi-square test.

Regarding referrals among pregnant women under the age of 15, only 0.5% received STD prophylaxis procedures; no pregnant woman underwent a semen collection procedure for DNA research or emergency contraceptive conduct; 30.9% were referred to the *Conselho Tutelar* (Guardianship Council); only 2.4% was sent to the *Delegacia de Proteção à Criança* (Child Protection Police); and 84.2% were referred to Primary Care (Table 5).

**Table 5:** Characteristics of referrals for pregnant women up to 14 years old and other pregnant women notified of sexual violence in Rio Branco (AC) from 2011 to 2016.

Characteristic		Pregnant women between 10 and 14 years old		Pregnant women aged 15 or over		p^a^
		n	%	n	(%)	
**STD prophylaxis**						
	Yes	3	0.5	9	7.3	0.000^a^
	No	637	99.5	115	92.7	
**Semen collection**						
	Yes	0	0.0	1	0.8	0.023^a^
	No	640	100.0	123	99.2	
**Emergency contraception**						
	Yes	0	0.0	4	3.2	0.001^a^
	No	637	100.0	120	96.8	
**Procedure for legal abortion**						
	Yes	4	0.6	5	4.1	0.007^a^
	No	633	99.4	116	95.9	
**Referral to women’s police station**						
	Yes	34	8.0	24	23.3	0.000^a^
	No	390	92.0	79	76.7	
**Referral guardianship advice**						
	Yes	123	30.9	20	19.6	0.024^a^
	No	275	69.1	82	80.4	
**Referral to the child protection police station**						
	Yes	10	2.4	1	1.0	0.700
	No	415	97.6	102	99.0	
**Referral to the Health sector**						
	Basic attention	320	84.2	72	75.8	0.053
	Hospitalization	60	15.8	23	24.2	

Missing or ignored values: STD prophylaxis: 0.7%; Semen collection: 0.6%; Emergency contraception: 0.9%; Procedure for legal abortion: 1.1%; Referral to the Women's Police Station: 16.2%; Referral to Guardianship Council: 17.1%; Referral to the Children’s Police Station: 15.9%; Health sector referral: 16.2%.

a p-value < 0.05 indicating statistically significant difference by the chi-square test.

## DISCUSSION

The distribution of SV notifications by year of study in the city of Rio Branco shows a higher concentration of cases in 2012 (18.7%) and 2013 (19.2%). These periods were cited in a study that analyzed the distribution of the SINAN notification rate (per 100,000 women) across the country from 2009 to 2013, wherein was found that the state of Acre presented the highest rates of registration in the country in the years 2011 (33.4), 2012 (54.5) and 2013 (70.9), compared to the others states^9^.

Among the facts that somehow contributed for the state of Acre to stand out in the period studied, we can mention the advance in the structuring of the specialized service to assist SV in the capital, with the MBH as a reference unit, which in 2010 started to have an outpatient clinic and multidisciplinary team for reception and follow-up of victims. The year 2011 was marked by training, meetings, and local debates on the topic, in addition to the construction of flows and protocols. The engagement of the team involved in the process and publication of Decree No. 104 determined the compulsory notification of the SV^10^ appeal, clarifying the difference in distribution between the years studied and the findings in the previous study^9^.

The notified SV victims presented a profile similar to that found in other studies, reinforcing the evidence on the vulnerability of the young female population to SV^9,11,12^. The general characteristics of the population of the municipality of Rio Branco collaborate with this profile, as its age base is composed of young people, with 29.20% of the population aged 15 years or less; many of them in situations of social vulnerability and poverty, a statement confirmed by the high number of teenage mothers, corresponding to 4.1% of women aged 10 to 17 years and unemployedpeople^13^.

In comparison to the profile of victims with other states in Brazil, the authors examined the SV against women, aged 10 years or more, in Santa Catarina, from 2008 to 2013. They observed a higher frequency of cases in the age group between 15 and 19 years old (83.7%)^14^. In turn, in Piauí state, between 2009 and 2016, the chance of SV prevailed among single girls aged 20 years or less, up to the 8th grade, with the intimate partner being the aggressor^15^.

When examining the place of occurrence of SV, for all age groups and at night, the records inform the victim’s residence as the main place - similar results were found in Recife (PE), with a predominance of women victims of SV in the age group from 10 to 19 years old (43.0%), mixed-race and with low education, being the residence the most common place of occurrence^16^. When the place of violence is the victim’s home, the literature shows that there is an association between SV and other types of violence, especially physical and psychological. This association builds the sad scenario of intra-family sexual abuse and domestic violence, marked by the victim’s financial dependence on the aggressor^16^.

However, we highlighted the low reference to physical violence associated with SV in all age groups in this work. Among the possible justifications, we assume that the authority exercised by the aggressor is sufficient to intimidate the victim; the victim’s lack of interaction with the interviewing health professional, leading to the omission of information; or even some failure in the technique of data collection and filling in the form^17^.

In this study the single aggressor prevailed, especially the intimate partner, with a statistically significant association. It is also worth noting that there was a variation in the aggressor’s relationship with the victim depending on the age group, with the unknown aggressor being the most mentioned in the age group of women aged 20 years or more. Likewise, in national and international surveys, the aggressor’s bond with the victim varies according to her age, pointing out that during childhood, women are attacked mainly by their family members, in adolescence by their boyfriends, partners, and ex-partners, and when adults are susceptible to SV by strangers^18–20^.

The predominance of the aggressor intimate partner in the age group from 10 to 14 years old contrasts with the finding in Londrina (PR), where this bond was not even mentioned for the group of girls between 10 and 14 years old. The authors studied the population of children and adolescents aged 0 to 14 years in the city. They identified that the aggressors were frequently family members, 7% being the father, 30.1% the stepfather, 21.5% other relatives, and 3.8% unknown^21^.

Although adult women aged 20 or over have been victims of SV by an unknown aggressor, what stands out is that the place of aggression is their residence. Data coincides with findings in other places, but contrasting with other studies, in which SV happens mainly on streets and is committed by unknowns^19,21^. This result is justified by the recognition of the living conditions of these people, usually residing in low-income housing, where houses are nearby and security requirements are absent, facilitating the invasion by strangers, who are often drunk (63%).

The most reported type of SV was rape followed by carnal conjunction, occurring in 85.7% of the recorded cases. Some authors argue that only cases with an immediate impact on health tend to be notified by the health sector, with underreporting of other forms of SV, such as cases of harassment, exploitation of minors, and trafficking in persons for sexual purposes, for example^18^. These crimes were little reported in Rio Branco; however, another study^22^ reveals that the North and Northeast regions have the largest number of trafficking routes for women and adolescents, both nationally and internationally, trafficking for sexual purposes mainly between the ages of 15 and 25 years. Therefore, it is difficult to scale the underreporting of SV in the municipality^22^.

Despite the health alerts, rape notifications do not seem to represent the reality, as the available data indicate that the police have an average of 3 times more rape records in their databases than SINAN^23^.

During analysis, we observed that the main consequence of SV was pregnancy, especially among victims aged 10 to 14 years. To better understand the profile of this group, a comparative examination was chosen between pregnant women aged 10 to 14 years and the others. The analysis revealed that the aggressor’s bond as an intimate partner reaches 87% among pregnant victims aged 10 to 14 years, with repeated violence occurring in the victim’s home, with no predominance of the association of SV with other types of violence, such as physical violence (12.9%), for example. Studies show that intimate partner violence is marked by physical, psychological, and forced intercourse in marriage. Association was not found in the data on the group of pregnants^17,24^.

The high number of pregnant notifications is related to the commitment of the epidemiological surveillance team of the MBH, which acts decisively in the compulsory notification of the crime of rape of the vulnerable, whereby identified an adolescent pregnant woman aged 14 years or less in the institution.

We assume child marriage in this group of pregnant, between 10 and 14 years old, because 41.2% of pregnant women declare themselves as married or in a stable relationship. From a legal perspective, Law No. 13,811, of 2019 modified the civil code and prohibited, without exception, the marriage of minors under 16 years of age. Thus, these teenagers live criminally with their alleged partners or aggressors. Prior to that law, marriage between young people under the age of 16 could occur when authorized by those responsible in order to “avoid the imposition or execution of a criminal penalty or in case of pregnancy”^25^.

It is noteworthy that Brazil has the largest number of cases of child marriage in Latin America and the fourth in the world. Law No. 13,811 is an important advance, but it is still timid when compared to other countries that allow the marriage of girls only after the age of 18, and often with the requirement of parental and state consent²^6^, according to the World Bank report Child marriage brings negative consequences, such as dropping out of school, financial dependence, teenage pregnancy, leaving many teenagers to domestic violence and marital rape^27^.

Another result that characterizes social and family acceptance in pregnant women aged 14 years or less is that almost all pregnant women (99.5%) did not receive prophylaxis for sexually transmitted infections (STIs), with no records of emergency contraception, which was identified in only 0.6% seek legal abortion services. This demonstrates that the motivation to seek the health service was a complication during pregnancy or for childbirth care, entailing the SV notification. Study by Souto et al.^12^ further enriches the theme by confirming that more than half (51.6%) of pregnant girls up to 13 years old reported for rape in Brazil in SINAN, in the period 2011-2015, had their partner or ex-partner as the aggressor and received low coverage of prophylactic procedures for STIs and emergency contraception^12^.

The small number of referrals leads to reflection on the role of health agents when identifying suspected abuse pregnancy, demonstrating that pregnant teenagers are not seen by the team as victims who need multidisciplinary follow-up aimed at the prevention and treatment of injuries resulting from the SV. This statement is reinforced by a study carried out with health professionals in Family Health units in a municipality in Pernambuco, which found that only 34.8% of professionals who identified suspected cases notified the responsible agencies. The fear against the aggressor, the respect for “coexistence rules” established by the local society, often violent communities, and the lack of information justify the underreporting^28^.

However, the results of the present investigation are limited because it is a retrospective study, with secondary SINAN data. In analyzing these data, the problem of underreporting should be considered. It is motivated by different factors, ranging from the low demand for victims by the health system to the non-reporting by some health professionals, who do not recognize crimes against sexual freedom and do not know of the mandatory notification. In addition to underreporting, another problem identified was the reliability of the information, which depends on correctly filling out the FIN, transcribing the information and feeding the database, not allowing extrapolating results and calculating SV rates against women in the municipality.

This work confirmed the susceptibility of young women from Rio Branco to SV, demonstrating the need for involvement of different spheres to public services, such as education, health, public security, and public prosecutors in the fight against SV. We expect that this article contributes to the SINAN as a surveillance strategy against SV, supporting the planning and evaluation of public health policies.
