# Supplementary material for: Sexual violence against women in the Western Amazon
Source: Rev Saude Publica. 2021 Nov 16;55:92. doi: 10.11606/s1518-8787.2021055003069 (PMC8621459; doi:10.11606/s1518-8787.2021055003069)
Supplement: Translation

# Translation: Violência sexual em mulheres na Amazônia Ocidental

**Authors:** Júlia Souza Santos Cargnin, Juliana Scholtão Luna, Débora Melo de Aguiar, Bárbara Teles Cameli Rodrigues, Aldir Alves de Azevedo, Rodrigo Pinheiro Silveira

**Affiliations:** I Rio Branco AC Brasil Universidade Federal do Acre. Programa de Pós-Graduação em Saúde Coletiva. Rio Branco, AC, Brasil; II Rio Branco AC Brasil Universidade Federal do Acre. Centro de Ciências da Saúde e do Desporto. Rio Branco, AC, Brasil; III Rio Branco AC Brasil Universidade Federal do Acre. Programa de Pós-Graduação em Saúde Coletiva. Rio Branco, AC, Brasil

**Keywords:** Maus-Tratos Infantis, Estupro, Gravidez na Adolescência, Violência contra a Mulher

## Abstract

**OBJETIVO:**

Caracterizar os casos de violência sexual sofridos por mulheres notificadas pelo Sistema de Informação de Agravos de Notificação no município de Rio Branco (AC) no período de 2011 a 2016.

**MÉTODOS:**

Estudo descritivo com base nas informações do Sistema de Informação de Agravos de Notificação. A população do estudo foi constituída por mulheres vítimas de violência sexual notificadas no município de Rio Branco (AC), de 2011 a 2016.

**RESULTADOS:**

Os resultados da investigação apresentam maior número de notificação durante o ano de 2012, se destacando entre as vítimas aquelas com idade entre 10 e 14 anos, solteiras, pardas e com 5 a 8 anos de formação educacional. O local de ocorrência mais notificado foi a residência, predominando agressor único, do sexo masculino e não alcoolizado.

**DISCUSSÃO:**

O grande número de notificações de gestantes na faixa etária de 10 a 14 anos corresponde à notificação compulsória do estupro de vulnerável, identificado no momento de realização do pré-natal ou parto.

**CONCLUSÃO:**

Confirmou-se a suscetibilidade à violência sexual de mulheres jovens de Rio Branco, levantando a problemática do casamento infantil e da gravidez na adolescência.

## INTRODUÇÃO

A violência sexual (VS) é um dos tipos de violência praticada contra as mulheres, sendo considerada uma das mais cruéis e persistentes ao longo da história. Definida como todo ato sexual ou tentativa de obtê-lo por meio da violência e contra a vontade da pessoa, independentemente do tipo de relacionamento interpessoal existente. Inclui, em sua definição, a agressão verbal, a obtenção de vantagem econômica, o casamento forçado, o assédio sexual e o estupro^1^.

Alguns autores afirmam que fatores como idade, escolaridade, renda, consumo de álcool e/ou drogas, estado civil e orientação sexual predispõem a vítima a esse tipo de violência. Todavia, as reais prevalências são de difícil obtenção em razão da subnotificação, seja por constrangimento ou medo da vítima e devido à variabilidade entre os estudos em relação às populações, instrumentos, condições de privacidade, treinamento das entrevistadoras, técnicas de coleta de informações e as definições de violência adotadas na metodologia^2^.

Apesar disso, indicadores confiáveis têm permitido constatar que a VS está entre as principais causas da redução de anos de vida saudável por incapacidades^3^. De acordo com Schraiber^4^, a VS segue um padrão comum, ocorrendo principalmente em ambientes privados, perpetrado por pessoas íntimas e que se repete de forma cíclica.

No Brasil, foram obtidos nas últimas décadas alguns avanços na estratégia de prevenção à VS contra as mulheres por meio das Delegacias Especializadas de Atendimento às Mulheres, Coordenadorias das Mulheres, Políticas de Enfrentamento à Violência e da Lei Maria da Penha.

Desde 2004, a VS e doméstica são de notificação compulsória pelos serviços de saúde. Contudo, apenas cinco anos depois, com o Sistema de Vigilância de Violências e Acidentes (VIVA), vinculado ao Sistema Informações de Agravo de Notificações (SINAN), a notificação da violência interpessoal e autoprovocada começa a ser realizada de forma incipiente, em unidades sentinelas dos municípios^5^. A partir de 2014, tanto a VS quanto a violência doméstica passam a ser de notificação imediata. Para tanto, passou-se a utilizar como instrumento padronizado para coleta de dados e registro o SINAN, o tornando um sistema importante de base de dados para estudos epidemiológicos e planejamento das ações em saúde^6^. Entretanto, é facultada à mulher a decisão quanto ao registro da ocorrência policial e da realização de exames periciais.

Mesmo levando em conta a subnotificação, os dados oficiais do Ministério da Saúde (MS) de 2011 indicavam 15,9 casos de VS por 100.000 mulheres, com variações conforme as regiões brasileiras examinadas, tendo influência os níveis socioeconômicos e educacionais da população^7^.

A escassez de estudos epidemiológicos que abordem a temática da VS contra mulheres na região Norte do país se dá, possivelmente, em decorrência da vasta extensão territorial e por possuir localidades de difícil acesso, algumas carentes de públicos serviços essenciais, somada às condições socioeconômicas precárias de parte considerável de sua população – além do mais, o desenvolvimento de estudos de prevalência são laboriosos e a subnotificação dos casos de VS é uma grave realidade^8^.

Esta situação não é diferente no município de Rio Branco, capital do estado do Acre, razão pela qual esse artigo busca caracterizar os casos de VS contra mulheres notificados no SINAN no município, tendo como recorte o período de 2011 a 2016.

## MÉTODOS

Trata-se de um estudo observacional do tipo descritivo, retrospectivo e de abordagem quantitativa, que analisou o perfil da VS contra mulheres notificadas nos serviços de saúde no período de 2011 a 2016 no município de Rio Branco (AC).

O estudo utilizou os dados do Sistema Informação de Agravos de Notificação (SINAN) referentes às notificações realizadas no município de Rio Branco por meio da Ficha de Notificação (FIN) de violência doméstica, sexual e/ou outras violências, cedidos pela Secretaria de Saúde Municipal.

Durante o período de realização da pesquisa, quase a totalidade das notificações, tanto do município quanto do estado, são realizadas na Maternidade Bárbara Heliodora (MBH), unidade pública de saúde situada na capital e referência no atendimento às vítimas de VS e a única cadastrada para procedimento de interrupção legal da gestação. A MBH presta assistência multiprofissional às mulheres vítimas de VS, conta com uma equipe capacitada para realização de procedimentos médicos e multidisciplinares e realiza os encaminhamentos necessários dentro da rede de apoio.

No período de 2011 a 2016, foram preenchidas 3.358 FIN de violência doméstica, sexual e/ou outras violências. Dessas, 1.648 casos corresponderam a VS contra mulheres com idade igual ou maior a 10 anos. A MBH foi responsável pela notificação de 1.529 casos, portanto, 92,8% das notificações registradas.

Para o presente estudo, foram selecionados na base de dados do SINAN todos os casos com registro da notificação de violência interpessoal e autoprovocada com o código genérico Y09, que corresponde às agressões por meio não especificado da Classificação Estatística Internacional de Doenças e Problemas Relacionados à Saúde (CID) – 10, além de selecionados pelo sexo feminino. Foram utilizados como critérios de inclusão dos casos a confirmação do preenchimento do campo “violência sexual” e a idade da vítima igual ou maior a 10 anos.

Portanto, a população de estudo para caracterização epidemiológica foi constituída por casos suspeitos ou confirmados de VS, pertencentes ao sexo feminino e com idade igual ou maior de 10 anos notificadas no município de Rio Branco no período de 1 de janeiro de 2011 a 31 de dezembro de 2016. Foram analisados os 1.648 casos que atenderam os critérios utilizados.

Também foram coletadas informações sociodemográficas, como faixa etária, situação conjugal, raça e escolaridade, referentes a condição da mulher, por exemplo, gestante e/ou com presença de deficiência ou transtorno; informações da violência, como local de ocorrência, horário, repetição dos casos, violência associada, tipo de penetração, número de envolvidos, sexo do provável agressor, suspeita do uso de álcool, vínculo com a vítima, lesões físicas e demais consequências; e encaminhamentos tomados, por exemplo, profilaxia DST, coleta de sêmen, contracepção de emergência, procedimento para aborto legal, encaminhamento pra a Delegacia da Mulher, encaminhamento para o Conselho Tutelar, encaminhamento para a Delegacia de Proteção à Criança e encaminhamento para o setor de saúde.

Vale ressaltar que, considerando a legislação nacional acerca do estupro de vulnerável (< 14 anos), foi realizada uma análise separada em faixas etárias, sendo classificada como: vulnerável as vítimas entre 0 a 14 anos; jovens as vítimas entre 15 a 19 anos; e adultos as vítimas entre 20 ou mais. Posteriormente, foi dicotomizada em gestantes com 10 a 14 anos e gestantes com 15 ou mais, identificando mais incidência de casos neste último grupo.

Para as variáveis categóricas foram obtidas frequências absolutas (n) e relativas (%), e as variáveis contínuas foram analisadas por meio das medidas de tendência central (média e mediana) e medidas de dispersão (desvio padrão). As diferenças estatísticas entre os grupos foram verificadas por meio do teste qui-quadrado, considerando um nível de significância de 5%. Para análise dos dados foi utilizado o programa Statistical Package for Social Science (SPSS) versão 20. O presente estudo respeita os princípios éticos de pesquisa com seres humanos, tendo sido aprovado pelo Comitê de Ética e Pesquisa do HCA/FUNDHACRE sob o parecer n° 2.127.216.

## RESULTADOS

A distribuição das notificações de VS contra mulheres do município de Rio Branco durante o período examinado apresenta uma maior concentração de casos entre os anos de 2012 (18,7%) e 2013 (19,2%) (dado não apresentado em tabela).

A Tabela 1 descreve o perfil epidemiológico das mulheres vítimas de VS entre 2011 e 2016, com média de idade da população de 15,56 anos, mediana de 16 anos, idade mínima de 10 e máxima de 65. Em relação a distribuição de frequências, a maior proporção das mulheres possuía entre 10 a 14 anos (70,8%), eram solteiras (68,5%), pardas (84,0%), apresentavam entre 5 a 8 anos de estudo (60,3%), estavam grávidas (51,8%), não apresentavam deficiência ou transtorno (95,3%) e moravam em Rio Branco (73,4%).

**Tabela 1:** Distribuição de frequências nas variáveis estudadas referentes à vítima, ao tipo de agressão e ao agressor em Rio Branco (AC) no período 2011 a 2016.

Variável		n	%
**Faixa etária**			
	10 a 14 anos	1.166	70,8
	15 a 19 anos	268	16,2
	20 anos ou mais	214	13,0
	Total	1.648	100,0
**Situação conjugal**			
	Solteira	1.061	68,5
	Casada/União estável	473	30,6
	Separada/Viúva	14	0,9
	Total	1.548	100,0
**Raça**			
	Branca	157	9,6
	Preta	76	4,6
	Parda	1.376	84,0
	Amarela/Indígena	30	1,8
	Total	1.639	100,0
**Escolaridade**			
	Até 4 anos de estudo	262	16,6
	5 a 8 anos de estudo	955	60,3
	9 a 11 anos de estudo	321	20,3
	12 anos ou mais de estudo	45	2,8
	Total	1.583	100
**Gestante**			
	Sim	771	51,8
	Não	717	48,2
	Total	1.488	100
**Presença de deficiência ou transtorno**			
	Sim	76	4,7
	Não	1.554	95,3
	Total	1.630	100
**Município de ocorrência**			
	Rio Branco	1.147	73,4
	Outros	416	26,6
	Total	1.563	100
**Local de ocorrência**			
	Residência	1.350	84,5
	Via pública	101	6,3
	Habitação coletiva	27	1,7
	Escola	10	0,6
	Comércio/Serviços	8	0,5
	Indústrias/Construção	7	0,4
	Bar ou similares	4	0,3
	Local de prática esportiva	2	0,1
	Outro/Motel	88	5,5
	Total	1.597	100
**Hora de ocorrência**			
	00h01–06h00	141	11,6
	06h01–12h00	189	15,6
	12h01–18h00	239	19,7
	18h01–24h00	645	53,1
	Total	1.214	100
**Ocorreu outras vezes**			
	Sim	1.049	66,0
	Não	541	34,0
	Total	1.590	100,0
**Violência associada**			
	Não	1.200	73,2
	Ameaça	26	1,6
	Força corporal	63	3,8
	Arma branca	14	0,9
	Enforcamento	4	0,2
	Arma de fogo	112	6,8
	Lesão por objeto pontiagudo	1	0,1
	Queimadura	1	0,1
	Mais de um tipo	204	12,4
	Outros	14	0,9
	Total	1.639	100
**Tipo de penetração**			
	Sem penetração	31	2,8
	Oral	10	0,9
	Anal	12	1,1
	Vaginal	949	85,7
	Mais de um tipo	105	9,5
	Total	1.107	100
**Número de envolvidos**			
	Um	1.538	94,0
	Dois ou mais	99	6,0
	Total	1.637	100
**Sexo do provável agressor**			
	Masculino	1.606	97,7
	Feminino	29	1,8
	Ambos os sexos	9	0,5
	Total	1.644	100,0
**Suspeita do uso de álcool**			
	Sim	415	26,3
	Não	1.164	73,7
	Total	1.579	100,0
**Vínculo com a vítima **			
	Parceiro íntimo	874	53,7
	Conhecido	315	19,3
	Familiar	103	6,3
	Desconhecido	266	16,3
	Outros	71	4,4
	Total	1.629	100,0

Quanto às características da ocorrência da violência, a residência foi o principal local em 84,5% dos casos. O principal turno de ocorrência foi o período da noite, entre às 18h01 e 24h, representando 53,1%. 66,0% dos casos eram reincidentes e 73,2% não associados a outra violência. Quanto ao ato violento e ao agressor, a maioria dos casos foi referida penetração vaginal (85,7%), apenas um agressor envolvido (94,0%), do sexo masculino (97,7%), sem suspeita quanto ao consumo de álcool (73,7%). O vínculo com a vítima foi principalmente o parceiro íntimo (53,7%).

A Tabela 2 retrata o perfil das vítimas de VS segundo características sociodemográficas de acordo com a faixa etária. Houve predomínio de mulheres na faixa etária de 10 a 14 anos, pardas, entre 5 e 8 anos de estudo e solteiras, apresentando variação estatística significante entre as faixas etárias.

**Tabela 2:** Características das mulheres vítimas de violência sexual por faixa etária, conforme características sociodemográficas, notificadas em Rio Branco (AC) no período de 2011 a 2016.

Característica		Total		10 a 14 anos		15 a 19 anos		20 ou mais		p
		n	(%)	n	(%)	n	(%)	n	(%)	
**Cor da pele**										
	Branca	157	9,6	85	7,3	36	13,5	36	17,0	0,000^a^
	Negra	76	4,6	53	4,6	12	4,5	11	5,2	
	Parda	1376	83,6	996	85,9	217	81,3	163	76,9	
	Amarela	5	0,3	3	0,3	0	0	2	0,9	
	Indígena	25	1,5	23	2,0	2	0,7	0	0,0	
**Escolaridade**										
	0–4	262	16,6	204	18,2	21	8,1	37	18,1	0,000^a^
	5–8	955	60,3	788	70,4	121	46,7	46	22,5	
	9–11	321	20,3	128	11,4	104	40,2	89	43,6	
	12 ou mais	45	2,8	0	0,0	13	5,0	32	15,7	
**Ocupação**										
	Estudante	665	66,0	495	71,3	141	74,2	29	23,4	0,000^a^
	Empregada	22	2,2	0	0,0	5	2,6	17	13,7	
	Outros	321	31,8	199	28,7	44	23,2	78	62,9	
**Situação Conjugal**										
	Solteira	1061	68,5	727	67,6	206	78,3	128	61,2	0,000^a^
	Casada/União estável	473	30,6	346	32,2	57	21,7	70	33,5	
	Separada	9	0,6	2	0,1	0	0,0	7	3,4	
	Viúva	5	0,3	1	0,1	0	0,0	4	1,9	

Valores ignorados ou em branco: Raça: 0,9%; Escolaridade: 1,9%; Ocupação: 19,1%; Situação conjugal: 1,1%.

a p-valor < 0,05 indicando diferença estatística significante pelo teste qui-quadrado.

A Tabela 3 apresenta as características do ato violento e do agressor conforme a faixa etária da vítima. Independentemente da idade, a residência foi o local de ocorrência mais notificado. A noite foi o principal turno de ocorrência, exceto entre as mulheres com 20 anos ou mais, em que a maior ocorrência foi durante a madrugada. A violência foi causada predominantemente pelo parceiro íntimo entre as mais jovens e por desconhecido entre mulheres com 20 anos ou mais. O ato violento foi praticado por apenas um agressor, mais de uma vez entre jovens com menor idade e sem história de uso de álcool pelo agressor. O principal tipo de penetração foi a vaginal, não havendo registros de lesões físicas. Contudo, o último caso esteve associado à agressão sexual em 18,2% dos registros, sendo mais frequente entre as mulheres de 20 anos ou mais. A gravidez foi a principal consequência entre as mais jovens, enquanto para as demais faixas etárias, não houve registros de consequências.

**Tabela 3:** Características da violência sexual segundo a agressão e o agressor nas diferentes faixas etárias de mulheres notificadas em Rio Branco (AC) no período de 2011 a 2016.

Característica		Total		10 a 14 anos		15 a 19 anos		20 ou mais		p
		n	%	n	(%)	n	(%)	n	(%)	
**Local da Ocorrência**										
	Residência	1350	84,5	1037	91,4	185	72,8	128	61,2	0,000^a^
	Via Pública	101	6,3	29	2,6	28	11,0	44	21,1	
	Outro	146	9,1	68	6,0	41	16,1	37	17,7	
**Turno da ocorrência**										
	Noite	645	53,1	494	60,4	91	45,0	60	30,9	0,000^a^
	Tarde	239	19,7	161	19,7	48	23,8	30	15,5	
	Manhã	189	15,6	125	15,3	28	13,9	36	18,6	
	Madrugada	141	11,6	38	4,6	35	17,3	68	35,1	
**Vínculo com o agressor**										
	Parceiro íntimo	874	53,7	761	66,1	91	34,3	22	10,4	0,000^a^
	Conhecido	315	19,3	198	17,2	61	23,0	56	26,4	
	Familiar	103	6,3	80	6,9	19	7,2	4	1,9	
	Desconhecido	266	16,3	60	5,2	82	30,9	124	58,5	
	Outros	71	4,4	53	4,6	12	4,5	6	2,8	
**Número de envolvidos**										
	Um	1538	94,0	1120	96,5	240	90,2	178	84,8	0,000^a^
	Dois ou mais	99	6,0	41	3,5	26	9,8	32	15,2	
**Violência de repetição**										
	Sim	1049	66,0	862	76,8	128	49,2	59	28,5	0,000^a^
	Não	541	34,0	261	23,2	132	50,8	148	71,5	
**Uso de álcool**										
	Sim	415	26,3	209	18,5	80	31,7	126	63,0	0,000^a^
	Não	1164	73,7	918	81,5	172	68,3	74	37,0	
**Tipo de penetração**										
	Sem penetração	31	2,8	25	3,3	2	1,0	4	2,7	0,000^a^
	Vaginal	949	85,7	675	89,5	177	86,8	97	65,1	
	Anal	12	1,1	5	0,7	3	1,5	4	2,7	
	Oral	10	0,9	3	0,4	3	1,5	4	2,7	
	Mais de um tipo	105	9,5	46	6,1	19	9,2	40	26,8	
**Lesões físicas**										
	Sim	200	18,2	107	14,2	39	18,9	54	38,0	0,000^a^
	Não	900	81,8	645	85,8	167	81,1	88	62,0	
**Consequências**										
	Nenhuma	481	43,1	243	31,9	117	56,8	121	81,2	0,000^a^
	DST	19	1,7	13	1,7	4	1,9	2	1,3	
	Gravidez	607	54,3	499	65,5	84	40,8	24	16,1	
	Suicídio	2	0,2	1	0,1	0	0,0	1	0,7	
	Duas ou mais	8	0,7	6	0,8	1	0,5	1	0,7	

Valores ignorados ou em branco: Local da ocorrência: 1,5%; Turno da ocorrência: 12,9%; Tipo de penetração: 16,1%; Violência de repetição: 1,7%; Consequência da violência: 15,8%; Lesão física associada: 16,3%; Número de envolvidos: 0,3%; Vinculo com o agressor: 0,6%; Uso de álcool pelo agressor: 2,1%.

a p-valor < 0,05 indicando diferença estatística significante pelo teste qui-quadrado.

Em relação às vítimas gestantes no momento da notificação da VS, independentemente da faixa etária, a maior proporção de casos eram mulheres solteiras, sendo a residência o principal local de ocorrência da violência, predominantemente no turno da noite, tendo como principal agressor o parceiro íntimo. A violência foi registrada como sendo de repetição, com maior ocorrência de penetração vaginal, não associada a lesão física e tendo a gravidez como principal consequência (Tabela 4).

**Tabela 4:** Características das mulheres gestantes vítimas de violência sexual segundo a agressão sofrida e o agressor para os estratos gestantes de até 14 anos e demais gestantes notificadas em Rio Branco (AC) no período de 2011 a 2016.

Característica		Gestantes entre 10 e 14 anos		Gestantes de 15 anos ou mais		p
		n	(%)	n	(%)	
**Situação conjugal**						
	Solteira	368	58,5	79	64,2	0,636
	Casada/União estável	259	41,2	44	35,8	
	Separada	1	0,2	0	0,0	
	Viúva	1	0,2	0	0,0	
**Local da ocorrência**						
	Residência	596	94,8	102	85,7	0,000^a^
	Via publica	6	1,0	7	5,9	
	Outros	27	4,3	10	8,4	
**Turno da ocorrência**						
	Noite	302	70,1	44	52,4	0,000^a^
	Tarde	70	16,2	18	21,4	
	Manhã	54	12,5	15	17,9	
	Madrugada	5	1,2	7	8,3	
**Vínculo com agressor**						
	Parceiro íntimo	556	87,0	75	60,5	0,000^a^
	Conhecido	61	9,5	14	11,3	
	Familiar	8	1,3	2	1,6	
	Desconhecido	7	1,1	29	23,4	
	Outros	7	1,1	4	3,2	
**Violência de repetição**						
	Sim	523	83,4	77	63,6	0,000^a^
	Não	104	16,6	44	36,4	
**Tipo de penetração**						
	Sem penetração	2	0,5	0	0,0	0,001^a^
	Vaginal	421	98,4	95	92,3	
	Anal	1	0,2	2	1,9	
	Mais de um tipo	4	0,9	6	5,8	
**Lesão física**						
	Sim	55	12,9	11	10,6	0,518
	Não	371	87,1	93	89,4	
**Consequência**						
	Nenhuma	23	5,4	12	11,7	0,069
	Gravidez	402	93,7	89	86,4	
	Suicídio	1	0,2	0	0,0	
	Duas ou mais	3	0,7	2	1,9	

Valores ignorados ou em branco: Situação conjugal: 1,1%; Local da ocorrência: 1,5%; Turno da ocorrência: 12,9%; Tipo de penetração: 16,1%; Violência de repetição: 1,7%; Consequência da violência: 15,8%; Lesão física associada: 16,3%; Número de envolvidos: 0,3%; Vínculo com o agressor: 0,6%; Uso de álcool pelo agressor: 2,1%.

a p-valor < 0,05 indicando diferença estatística significante pelo teste qui-quadrado.

Em relação aos encaminhamentos entre gestantes com idade inferior a 15 anos, apenas 0,5% receberam condutas para profilaxia de DST; nenhuma gestante passou por procedimento de coleta de sêmen para pesquisa de DNA ou conduta contraceptiva de emergência; 30,9% foram encaminhadas para o Conselho Tutelar; apenas 2,4% foi encaminhada à Delegacia de Proteção à Criança e ao Adolescente; e 84,2% foram encaminhadas para Atenção Básica (Tabela 5).

**Tabela 5:** Características dos encaminhamentos para os estratos gestantes de até 14 anos e demais gestantes notificadas por violência sexual em Rio Branco (AC) no período de 2011 a 2016.

Característica		Gestantes entre 10 e 14 anos		Gestantes de 15 anos ou mais		p^a^
		n	%	n	(%)	
**Profilaxia DST**						
	Sim	3	0,5	9	7,3	0,000^a^
	Não	637	99,5	115	92,7	
**Coleta de sêmen**						
	Sim	0	0,0	1	0,8	0,023^a^
	Não	640	100,0	123	99,2	
**Contracepção de emergência**						
	Sim	0	0,0	4	3,2	0,001^a^
	Não	637	100,0	120	96,8	
**Procedimento para aborto legal**						
	Sim	4	0,6	5	4,1	0,007^a^
	Não	633	99,4	116	95,9	
**Encaminhamento delegacia da mulher**						
	Sim	34	8,0	24	23,3	0,000^a^
	Não	390	92,0	79	76,7	
**Encaminhamento conselho tutelar**						
	Sim	123	30,9	20	19,6	0,024^a^
	Não	275	69,1	82	80,4	
**Encaminhamento delegacia de proteção à criança**						
	Sim	10	2,4	1	1,0	0,700
	Não	415	97,6	102	99,0	
**Encaminhamento setor de saúde**						
	Atenção básica	320	84,2	72	75,8	0,053
	Internação	60	15,8	23	24,2	

Valores ausentes ou ignorados: Profilaxia DST: 0,7%; Coleta de sêmen: 0,6%; Contracepção de emergência: 0,9%; Procedimento para aborto legal: 1,1%; Encaminhamento Delegacia da Mulher: 16,2%; Encaminhamento Conselho Tutelar: 17,1%; Encaminhamento Delegacia da Criança: 15,9%; Encaminhamento setor de saúde: 16,2%.

a p-valor < 0,05 indicando diferença estatística significante pelo teste qui-quadrado.

## DISCUSSÃO

A distribuição das notificações de VS por ano de estudo do município de Rio Branco mostra uma maior concentração de casos em 2012 (18,7%) e 2013 (19,2%). Esses períodos foram citados em um estudo que analisou a distribuição da taxa de notificações do SINAN (por 100 mil mulheres) de todo o país no período de 2009 a 2013, onde foi constatado que, em comparação com os demais, o estado do Acre apresentou as maiores taxas de registro do país nos anos de 2011 (33,4), 2012 (54,5) e 2013 (70,9)^9^.

Dentre os fatos que de alguma forma contribuíram para o estado do Acre ter destaque no período estudado, podemos citar o avanço da estruturação do serviço especializado de atendimento às VS na capital, tendo a MBH como unidade referência, que em 2010 passou a contar com um ambulatório e equipe multidisciplinar para acolhimento e seguimento das vítimas. O ano de 2011 foi marcado por treinamentos, reuniões e debates locais sobre o tema, além da construção de fluxos e protocolos. O engajamento da equipe envolvida no processo, somada a publicação da portaria n° 104 que determinou a notificação compulsória do agravo de VS^10^, esclarece a diferença da distribuição entre os anos estudados e os achados no estudo anterior^9^.

As vítimas de VS notificadas apresentaram o perfil semelhante ao encontrado em outros estudos, reforçando as evidências sobre a vulnerabilidade da população jovem feminina à VS^9,11,12^. As características gerais da população do município de Rio Branco colaboram com este perfil, pois sua base etária é composta por jovens, sendo 29,20% da população com 15 anos ou menos; muitos deles em situação de vulnerabilidade social e pobreza, afirmação ratificada pelo elevado número de mães adolescentes, correspondendo a 4,1% das mulheres entre 10 a 17 anos e de pessoas desocupadas^13^.

Na comparação do perfil das vítimas com outros Estados do Brasil, os autores examinaram a VS contra mulheres de 10 anos ou mais em Santa Catarina no período de 2008 a 2013, onde observou maior frequência de casos na faixa etária entre 15 a 19 anos (83,7%)^14^. Por sua vez, no Piauí, entre 2009 a 2016, a chance de VS prevaleceu entre meninas com idade de 20 anos ou menos, até a 8ª série, solteiras, sendo o parceiro íntimo o agente da agressão^15^.

No exame do local de ocorrência da VS, para todas as faixas etárias e no horário noturno, os registros informam a residência da vítima como principal local – resultado semelhantes foram encontrados em Recife (PE), com predomínio de mulheres vítimas de VS na faixa etária de 10 a 19 anos (43,0%), pardas e de baixa escolaridade, sendo a residência o local mais comum das ocorrências^16^. Quando o local da violência é a residência da vítima, a literatura demonstra que há associação da VS com outras violências, principalmente com a física e psicológica. Essa associação constrói o triste cenário do abuso sexual intrafamiliar e da violência doméstica, marcado pela dependência financeira da vítima com o agressor^16^.

Entretanto, destacou-se nesse trabalho a baixa referência à violência física associada à VS em todas as faixas etárias. Entre as possíveis justificadas se encontram o fato da autoridade exercida pelo agressor ser o suficiente para intimidar a vítima; a falta de interação da vítima com o profissional de saúde entrevistador, levando a omissão de informações; ou, ainda, alguma falha na técnica de coleta de dados e preenchimento da ficha^17^.

Nas investigações realizadas neste estudo, prevaleceu o agressor único, principalmente o parceiro íntimo, com uma associação estatisticamente significativa. Vale ainda destacar que houve variação da relação do agressor com a vítima a depender da faixa etária, sendo o agressor desconhecido o mais citado na faixa etária de mulheres de 20 anos ou mais. Da mesma forma, em pesquisas nacionais e internacionais o vínculo do agressor com a vítima varia conforme a idade dela, apontando que durante a infância as mulheres são agredidas principalmente por seus familiares, na adolescência por seus namorados, parceiros e ex-parceiros e quando adultas estão suscetíveis à VS por desconhecidos^18–20^.

A predominância do agressor parceiro íntimo na faixa etária de 10 a 14 anos contrasta com o achado em Londrina (PR), onde esse vínculo sequer foi citado para o grupo de meninas entre 10 a 14 anos. Os autores estudaram a população de crianças e adolescentes de 0 a 14 anos do município e identificaram que os agressores eram, na maioria das vezes, membros da família, sendo 7% o pai, 30,1% o padrasto, 21,5% outros parentes e, em menor proporção, 3,8% desconhecidos^21^.

Ainda que as mulheres adultas com 20 ou mais tenham sido vítimas de VS por agressor desconhecido, o que mais chama a atenção é o local da agressão ser a residência. Esse dado coincide com o encontrado em outros locais, contrastando, porém, com outros estudos nos quais a VS por desconhecido acontece principalmente na via pública^19,21^. Justifica-se esse resultado por meio do reconhecimento das condições de moradia dessas pessoas, geralmente residentes em habitações populares, onde existe proximidade entre as casas e ausência dos mínimos requisitos de segurança, facilitando a invasão por estranhos, muitas vezes embriagados (63%).

O tipo de VS mais notificado foi o estupro seguido por conjunção carnal, ocorrendo em 85,7% dos casos registrados. Alguns autores argumentam que apenas os casos com impacto imediato à saúde tendem a ser notificados pelo setor de saúde, ocorrendo a subnotificação das outras formas de VS, como os casos de assédio, exploração de menor e tráfico de pessoas para fins sexuais, por exemplo^18^. Esses crimes foram pouco notificados em Rio Branco; entretanto, outro estudo^22^ revela que as regiões Norte e Nordeste possuem o maior número de rotas de tráfico de mulheres e adolescentes, tanto em âmbito nacional quanto internacional, sendo que o tráfico para fins sexuais ocorre principalmente nas idades entre 15 e 25 anos. Sendo assim, é difícil dimensionar a subnotificação da VS no município^22^.

Mesmo a notificação do crime de estupro pelo setor de saúde, ela parece não representar a realidade, visto que os dados disponíveis indicam que a polícia têm em média 3 vezes mais registros de estupro em suas bases de dados do que o SINAN^23^.

Durante a análise dos dados, observou-se que a principal consequência da VS foi a gestação, principalmente entre as vítimas de 10 a 14 anos. A fim de entender melhor o perfil desse grupo, optou-se por um exame comparativo entre as gestantes de 10 a 14 anos e as demais. A análise revelou que o vínculo do agressor como parceiro íntimo chega a 87% entre as vítimas gestantes de 10 a 14 anos, ocorrendo a violência de repetição, na residência da vítima, não havendo predomínio da associação da VS com outras violências, como a física (12,9%), por exemplo. Os estudos mostram que a violência pelo parceiro íntimo é marcada pela violência física, psicológica e a relação sexual forçada no casamento. Associação não foi encontrada nos dados sobre o grupo de gestantes^17,24^.

O elevado número de notificações de gestantes nessa faixa se deve ao empenho da equipe da vigilância epidemiológica da MBH, que, ao identificar uma gestante adolescente de idade menor ou igual a 14 anos internada na instituição, atua de forma contundente na notificação compulsória do crime de estupro de vulnerável.

A suposição do casamento infantil nesse grupo de gestantes entre 10 e 14 anos sustenta-se no dado de 41,2% das gestantes se declararem com *statu*s civil de casada ou em união estável. Sob a perspectiva jurídica, a Lei n° 13.811, de 2019, modificou o código civil e proibiu, sem exceções, o casamento de menor de 16 anos. Desta forma, essas adolescentes vivem de forma delituosa com seus supostos parceiros ou agressores. Anterior a essa lei, o matrimônio entre jovens com idade inferior a 16 anos poderia ocorrer quando autorizado pelos responsáveis a fim de “evitar imposição ou cumprimento de pena criminal ou em caso de gravidez”^25^.

Vale ressaltar que o Brasil tem o maior número de casos de casamento infantil da América Latina e o quarto no mundo. A Lei n° 13.811 é um importante avanço, mas ainda tímido quando comparado a outros países que, conforme o relatório do Banco Mundial, permitem o casamento de meninas apenas após os 18 anos e, muitas vezes, com a exigência do consentimento parental e do estado²^6^. O casamento infantil traz consequências negativas, como o abandono escolar, dependência financeira, gravidez na adolescência, abandonando muitas adolescentes à violência doméstica e ao estupro marital^27^.

Outro resultado que caracteriza a aceitação social e familiar em gestantes de 14 anos ou menos é que quase a totalidade das gestantes (99,5%) não recebeu profilaxia para infecções sexualmente transmissíveis (IST), não havendo registros de contracepção de emergência, identificado em apenas 0,6% a busca por serviços de aborto legal. Isso demonstra que a motivação para procurar o serviço de saúde foi uma intercorrência na gestação ou para assistência ao parto, quando é então notificada a VS. Estudo de Souto et al.^12^ enriquece ainda mais o tema ao confirmar que mais da metade (51,6%) das meninas gestantes de até 13 anos notificadas por estupro no Brasil no SINAN, no período de 2011 a 2015, tinham o parceiro ou ex-parceiro íntimo como agressor e receberam baixa cobertura de procedimentos profiláticos para IST e contracepção de emergência^12^.

O pequeno número de encaminhamentos leva a refletir sobre o papel dos agentes de saúde ao identificarem a gestação suspeita de abuso, demonstrando que as adolescentes gestantes não são vistas pela equipe como vítimas que necessitem de acompanhamento multidisciplinar voltado à prevenção e ao tratamento dos agravos decorrentes da VS. Essa afirmação pode ser sustentada por um estudo realizado com profissionais de saúde nas unidades de Saúde da Família em um município de Pernambuco, que se observou que apenas 34,8% dos profissionais que identificaram casos suspeitos notificaram os órgãos responsáveis. A ausência de notificação foi justificada pelo medo de represália por parte do agressor, respeito a “regras de convivência” estabelecidas pela sociedade local, muitas vezes comunidades violentas, e o não entendimento da obrigatoriedade da saúde com a notificação^28^.

Contudo, os resultados da presente investigação são limitados por se tratar de um estudo retrospectivo, com dados secundários SINAN. Na análise desses dados deve-se considerar o problema da subnotificação, motivada por diferentes fatores que vão desde a baixa procura das vítimas pelo sistema de saúde a não notificação por parte de alguns profissionais de saúde que não reconhecem os crimes contra a liberdade sexual e não sabem da obrigatoriedade da notificação. Além da subnotificação, outro problema identificado foi a confiabilidade da informação, que depende do preenchimento correto da FIN, transcrição das informações e alimentação do banco de dados, não permitindo extrapolar resultados e calcular taxas da VS contra a mulher no município.

Este trabalho confirmou a suscetibilidade de mulheres jovens de Rio Branco à VS, demonstrando a necessidade de envolvimento de diferentes esferas do serviço público, como educação, saúde, segurança pública e ministério público no combate à VS. Espera-se que esse artigo possa contribuir no sentido de reforçar o potencial do SINAN como estratégia de vigilância da VS, subsidiando o planejamento e a avaliação das políticas públicas de saúde.
